# Harmonized Segmentation of Neonatal Brain MRI

**DOI:** 10.3389/fnins.2021.662005

**Published:** 2021-05-25

**Authors:** Irina Grigorescu, Lucy Vanes, Alena Uus, Dafnis Batalle, Lucilio Cordero-Grande, Chiara Nosarti, A. David Edwards, Joseph V. Hajnal, Marc Modat, Maria Deprez

**Affiliations:** ^1^Centre for the Developing Brain, School of Biomedical Engineering and Imaging Sciences, King's College London, London, United Kingdom; ^2^Biomedical Engineering Department, School of Biomedical Engineering and Imaging Sciences, King's College London, London, United Kingdom; ^3^Department of Child and Adolescent Psychiatry, Institute of Psychiatry, Psychology and Neuroscience, King's College London, London, United Kingdom; ^4^Department of Forensic and Neurodevelopmental Science, Institute of Psychiatry, Psychology and Neuroscience, King's College London, London, United Kingdom; ^5^Biomedical Image Technologies, ETSI Telecomunicación, Universidad Politécnica de Madrid & CIBER-BNN, Madrid, Spain

**Keywords:** deep learning, segmentation, neonatal brain, unsupervised domain adaptation, cortical thickness

## Abstract

Deep learning based medical image segmentation has shown great potential in becoming a key part of the clinical analysis pipeline. However, many of these models rely on the assumption that the train and test data come from the same distribution. This means that such methods cannot guarantee high quality predictions when the source and target domains are dissimilar due to different acquisition protocols, or biases in patient cohorts. Recently, unsupervised domain adaptation techniques have shown great potential in alleviating this problem by minimizing the shift between the source and target distributions, without requiring the use of labeled data in the target domain. In this work, we aim to predict tissue segmentation maps on *T*_2_-weighted magnetic resonance imaging data of an unseen preterm-born neonatal population, which has both different acquisition parameters and population bias when compared to our training data. We achieve this by investigating two unsupervised domain adaptation techniques with the objective of finding the best solution for our problem. We compare the two methods with a baseline fully-supervised segmentation network and report our results in terms of Dice scores obtained on our source test dataset. Moreover, we analyse tissue volumes and cortical thickness measures of the harmonized data on a subset of the population matched for gestational age at birth and postmenstrual age at scan. Finally, we demonstrate the applicability of the harmonized cortical gray matter maps with an analysis comparing term and preterm-born neonates and a proof-of-principle investigation of the association between cortical thickness and a language outcome measure.

## 1. Introduction

Medical image deep learning has made incredible advances in solving a wide range of scientific problems, including tissue segmentation or image classification (Miotto et al., 2018). However, one major drawback of these methods is their applicability in a clinical setting, as many models rely on the assumption that the source and target domains are drawn from the same distribution. As a result, the efficiency of these models may drop drastically when applied to images which were acquired with acquisition protocols different than the ones used to train the models (Kamnitsas et al., 2017; Orbes-Arteaga et al., 2019).

At the same time, combining imaging data from multiple studies and sites is necessary to increase the sample size and thereby the statistical power of neuroimaging studies. However, one major challenge is the lack of standardization in image acquisition protocols, scanner hardware, and software. Inter-scanner variability has been demonstrated to affect measurements obtained for downstream analysis such as voxel-based morphometry (Takao et al., 2011), and lesion volumes (Shinohara et al., 2017). Therefore, the purpose of harmonizing magnetic resonance imaging (MRI) datasets is to make sure that the differences arising from different image acquisition protocols do not affect the analysis performed on the combined data. For example, volumetric and cortical thickness measures should only be affected by brain anatomy and not the acquisition protocol or scanners.

A class of deep learning methods called domain adaptation (DA) techniques aims to address this issue by suppressing the domain shift between the training and test distributions. In general, DA approaches are either semi-supervised, which assume the existence of labels in the target dataset, or unsupervised, which assume the target dataset has no labels. For example, a common approach is to train a model on source domain images and fine-tune it on target domain data (Ghafoorian et al., 2017; Kushibar et al., 2019). Although these methods can give good results, they can become impractical as more often than not the existence of labels in the target dataset is limited or of poor quality. Unsupervised domain adaptation techniques (Ganin and Lempitsky, 2015; Kerfoot et al., 2019) offer a solution to this problem by minimizing the disparity between a source and a target domain, without requiring the use of labeled data in the target domain.

In our previous work (Grigorescu et al., 2020), we investigated two unsupervised DA methods with the aim of predicting brain tissue segmentations on 2D axial slices of *T*2-weighted (*T*2w) MRI data of an unseen neonatal population. We proposed an additional loss term in one of the methods, in order to constrain the network to more realistic reconstructions. Our models were trained using as source domain a dataset with majority of term-born neonates and as target domain a preterm-only population acquired with a different protocol. We calculated mean cortical thickness measures for every subject in the two datasets and we performed an ANCOVA analysis in order to find group differences between the predicted source and target domains. This analysis showed that our proposed method achieved harmonization of our two datasets in terms of cortical gray matter tissue segmentation maps. In this paper, we build on the aforementioned framework, which we expanded in three main ways. First, we build and train 3D neural networks in order to capture more information about the neonatal brain. Second, we extend the validation of our trained models to subsets of the two cohorts matched for gestational age (GA) at birth and postmenstrual age (PMA) at scan, for which we analyse tissue volumes and global and local cortical thickness (CT) measures. Finally, we perform an analysis comparing term and preterm-born neonates on the harmonized cortical gray matter maps and we show the importance of harmonizing the data by a proof-of-principle investigation of the association between cortical thickness and a language outcome measure.

## 2. Materials and Methods

### 2.1. Data Acquisition and Preprocessing

The *T*2w MRI data used in this study was collected as part of two independent projects: the developing Human Connectome Project (dHCP^1^, approved by the National Research Ethics Committee REC: 14/Lo/1169), and the Evaluation of Preterm Imaging (ePrime^2^, REC: 09/H0707/98) study. The dHCP neonates were scanned during natural unsedated sleep at the Evelina London Children's Hospital between 2015 and 2019. The ePrime neonates were scanned at the neonatal intensive care unit in Hammersmith Hospital between 2010 and 2013 (Edwards et al., 2018). Infants with major congenital malformations were excluded from both cohorts.

The dHCP data was acquired using a Philips Achieva 3T scanner and a 32-channels neonatal head coil (Hughes et al., 2017), using a *T*2w turbo spin echo (TSE) sequence with fat suppression, and using the following parameters: repetition time *T*_*R*_ = 12 s, echo time *T*_*E*_ = 156 ms, TSE factor 12, and SENSE factors of 2.11 for the axial plane and 2.58 for the sagittal plane. Images were acquired with an in-plane resolution of 0.8 × 0.8 mm, slice thickness of 1.6 mm and overlap of 0.8 mm. For each volume, there was an acquisition of 125 slices in the transverse plane and 134 slices in the saggital plane. All data was motion corrected (Kuklisova-Murgasova et al., 2012; Cordero-Grande et al., 2018) and super-resolution reconstructed to a 0.5 mm isotropic resolution (Makropoulos et al., 2018).

The ePrime dataset was acquired with a Philips Intera 3T system and an 8-channel phased array head coil, using a *T*2w TSE sequence with parameters: repetition time *T*_*R*_ = 8.67 s, echo time *T*_*E*_ = 160 ms, and TSE factor 16. Images were acquired with an in-plane resolution of 0.86 × 0.86 mm, slice thickness of 2 mm and overlap of 1 mm. For each volume, the acquisition ranged between 92 and 106 slices in the transverse plane.

**Figure 1:**
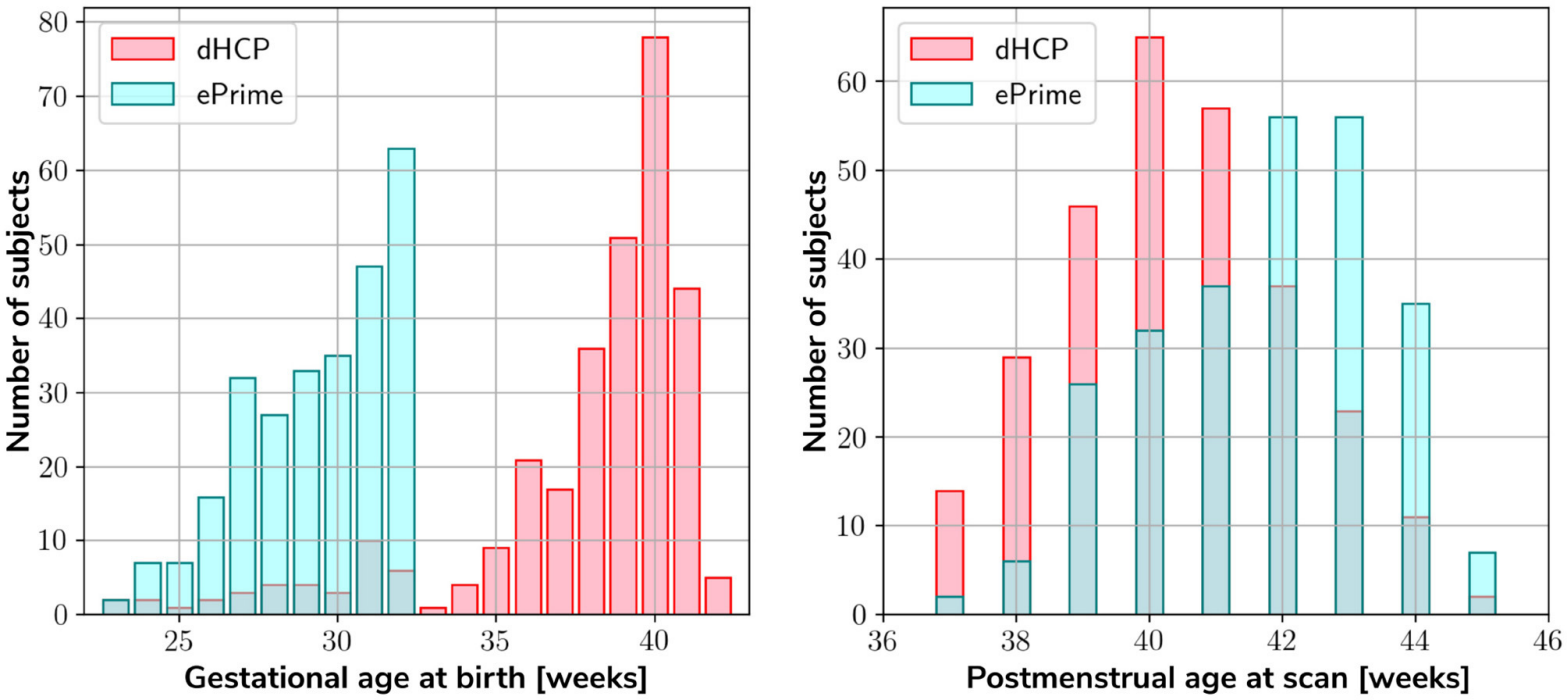
Age distribution of the subjects in our datasets, showing both their GA at birth, as well as their PMA at scan.

Our two datasets comprise of 403 MRI scans of infants (184 females and 219 males) born between 23 and 42 weeks GA at birth and scanned at term-equivalent age (after 37 weeks PMA) as part of the dHCP pipeline, and a dataset of 486 MRI scans of infants (245 females and 241 males) born between 23 and 33 weeks GA and scanned at term-equivalent age as part of the ePrime project. Figure 1 shows their age distribution.

Both datasets were pre-processed prior to being used by the deep learning algorithms. The ePrime volumes were linearly upsampled to 0.5 mm isotropic resolution to match the resolution of our source (dHCP) dataset. Both dHCP and ePrime datasets were rigidly aligned to a common 40 weeks gestational age atlas space (Schuh et al., 2018) using the MIRTK (Rueckert et al., 1999) software toolbox. Then, skull-stripping was performed on all of our data using the brain masks obtained with the Draw-EM pipeline for automatic brain MRI segmentation of the developing neonatal brain (Makropoulos et al., 2018). Tissue segmentation maps were obtained using the same pipeline (Draw-EM) for both (dHCP and ePrime) cohorts.

To train our networks, we split our datasets into 80% training, 10% validation, and 10% test (see Table 1), keeping both the distribution of ages at scan and the male-to-female ratio as close to the original as possible. We used the validation sets to keep track of our models' performance during training, and the test sets to report our final models' results and showcase their capability to generalize.

**Table 1:** Number of scans in different datasets used for training, validation and testing the models, together with their mean GA and PMA.

**Dataset**	**#Subjects**	**GA at birth [weeks]**	**PMA at scan [weeks]**
Train dHCP	340 (160♀ + 180♂)	39.1 (±2.7)	40.7 (±1.7)
Validate dHCP	32 (12♀ + 20♂)	39.3 (±1.6)	40.7 (±1.8)
Test dHCP	30 (12♀ + 19♂)	30 (±2.4)	41.4 (±1.7)
Train ePrime	417 (214♀ + 203♂)	29.6 (±2.3)	42.9 (±2.6)
Validate ePrime	38 (18♀ + 20♂)	29.8 (±2.3)	43 (±2.6)
Test ePrime	30 (13♀ + 18♂)	30 (±2.4)	41.4 (±1.7)

### 2.2. Unsupervised Domain Adaptation Models

**Figure 2:**
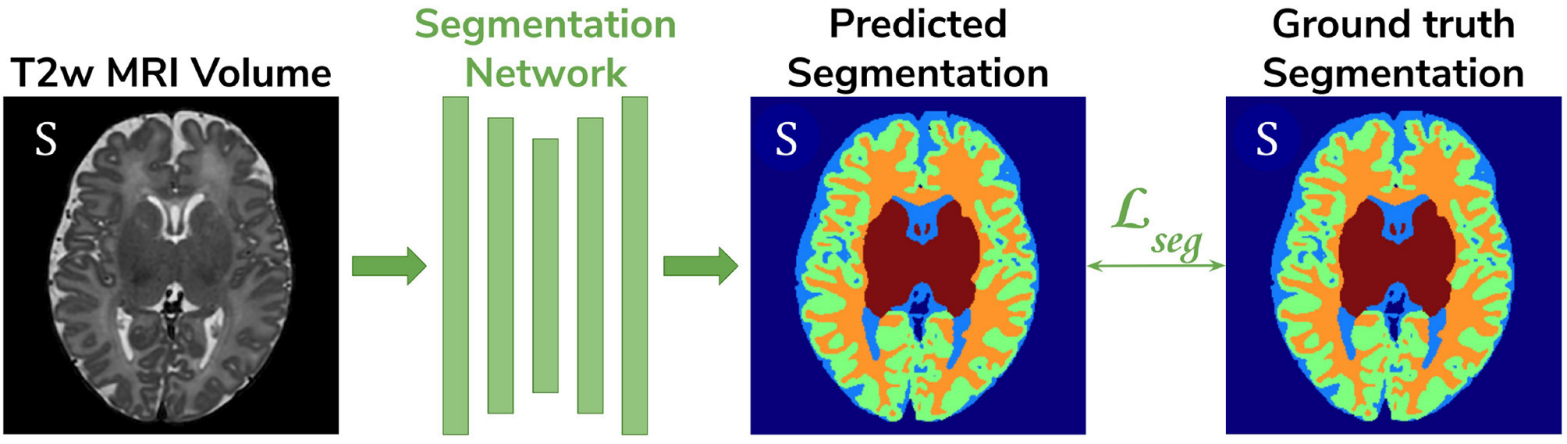
The baseline model consists of a 3D U-Net trained to segment source (dHCP) volumes. The input *T*2w MRI images, the predicted segmentation and the Draw-EM output segmentations are marked with S as they all belong to the source (dHCP) dataset.

**Figure 3:**
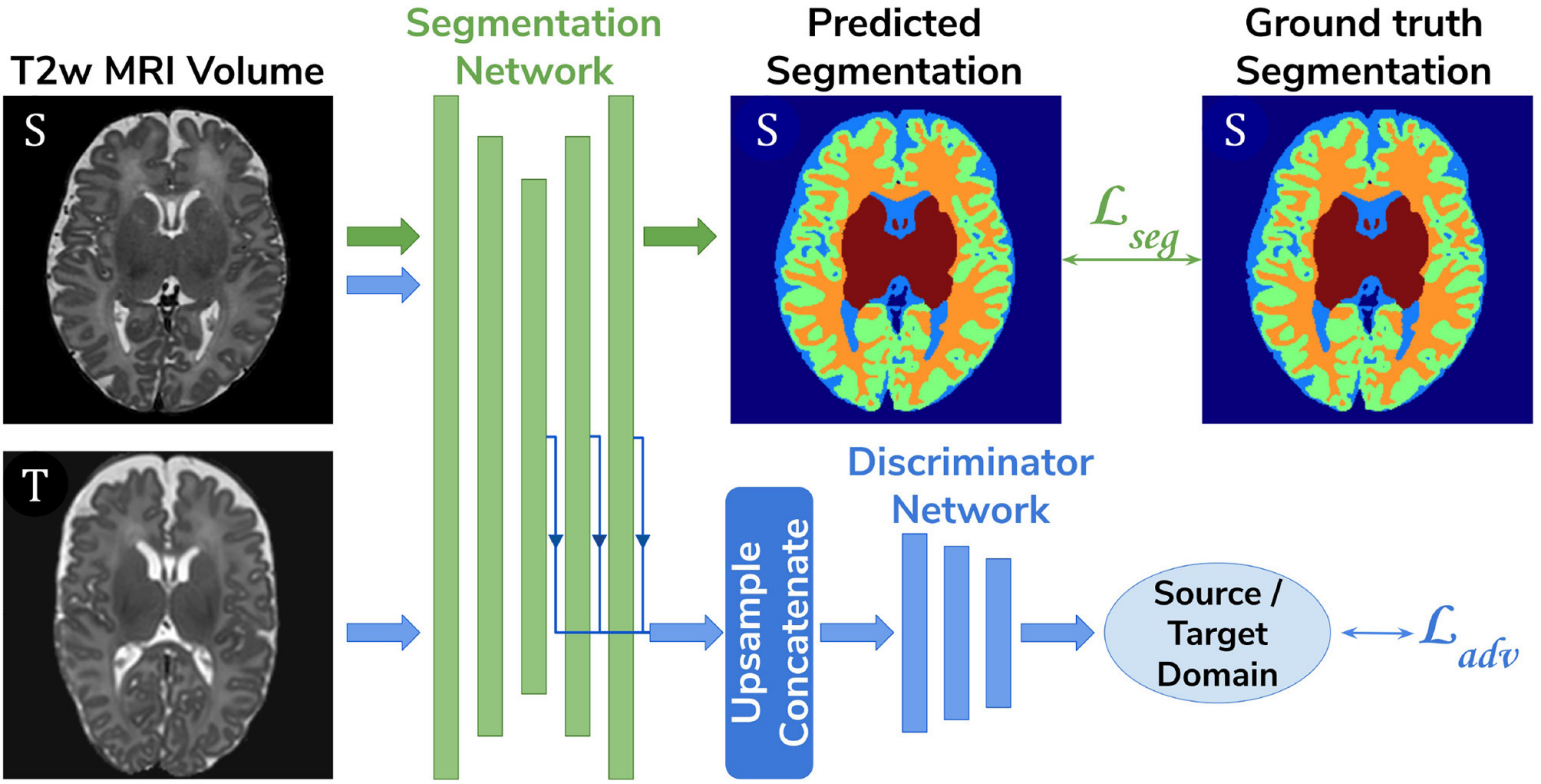
The latent space domain adaptation setup consists of a 3D U-Net trained to segment the source (dHCP) *T*2w MRI volumes, coupled with a discriminator network which forces the segmentation network to learn domain-invariant features. Both source (dHCP) and target (ePrime) images are fed to the segmentation network, but only source (dHCP) Draw-EM output labels are used to compute the segmentation loss. Source domain images are marked with S, while target domain images are marked with T, respectively.

**Figure 4:**
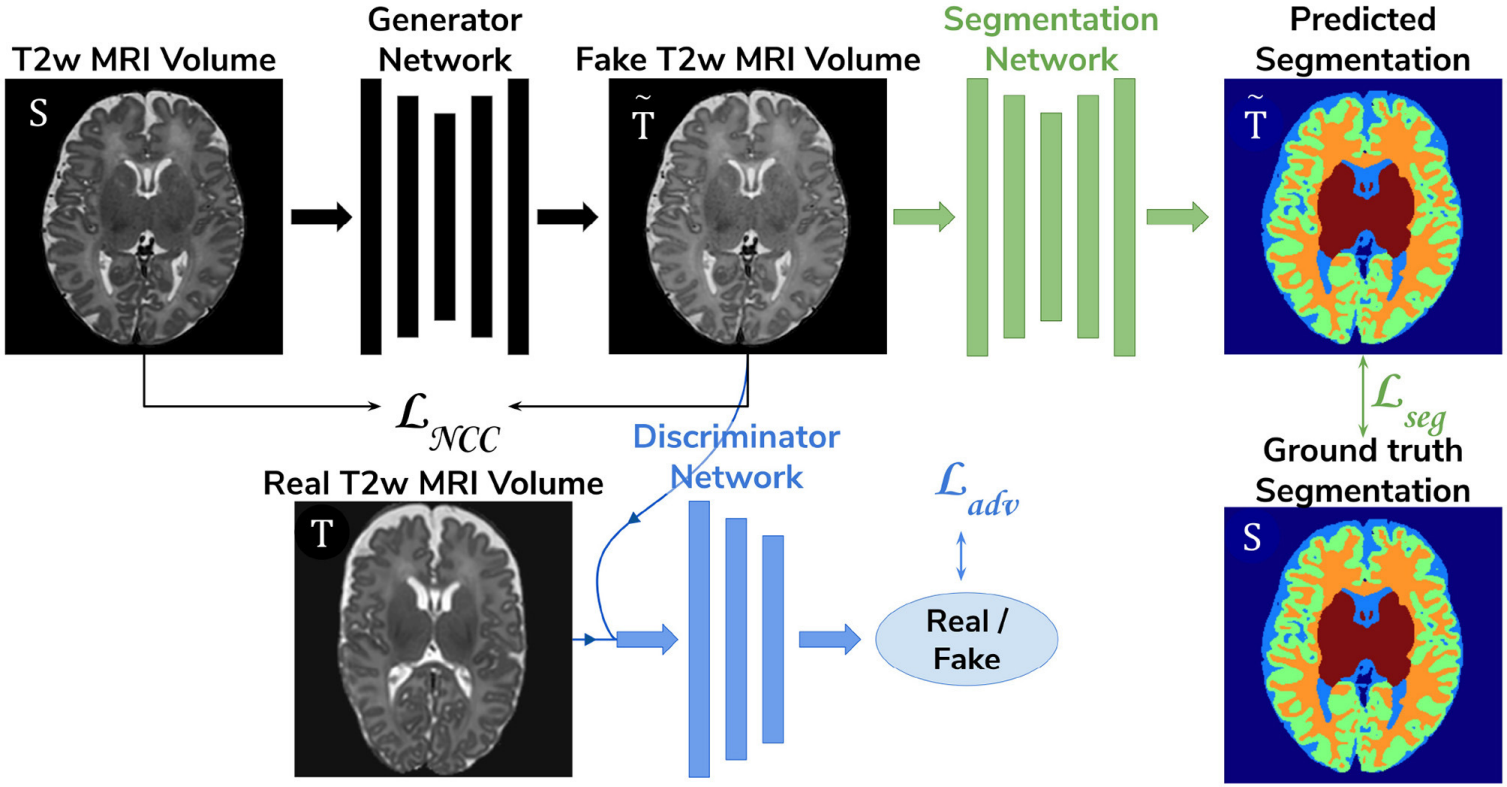
The image space domain adaptation setup uses a generator network to produce ePrime-like *T*2w MRI images (marked with $\tilde{\text{T}}$), which are then used as input into the segmentation network. The discriminator is trained to distinguish between real (ePrime) and synthesized (ePrime-like) volumes, while the generator is trained to produce realistic images in order to fool the discriminator. The normalised cross correlation (NCC) loss enforces image similarity between real and synthesized volumes.

To investigate the best solution for segmenting our target dataset (ePrime), we compared three independently trained deep learning models:

- **Baseline**. A 3D U-Net (Çiçek et al., 2016) trained on the source dataset (dHCP) only and used as a baseline segmentation network (see Figure 2).
- **Adversarial domain adaptation in the latent space**. A 3D U-Net segmentation network trained on source (dHCP) volumes, coupled with a discriminator trained on both source (dHCP) and target (ePrime) datasets (see Figure 3). This solution is similar to the one proposed by Kamnitsas et al. (2017) where the aim was to train the segmentation network such that it becomes agnostic to the data domain.
- **Adversarial domain adaptation in the image space**. Two 3D U-Nets, one acting as a generator, and a second one acting as a segmentation network, coupled with a discriminator trained on both real and synthesized ePrime volumes. The segmentation network is trained to produce tissue maps of the synthesized ePrime volumes created by the generator (see Figure 4). The normalized cross correlation (NCC) loss is added to the generator network to enforce image similarity between real and synthesized images, a solution which was previously proposed by Grigorescu et al. (2020).

To further validate the harmonized tissue maps, we trained an additional network (a 3D U-Net) to segment binary cortical tissue maps into 11 cortical substructures (see Table 2) based on anatomical groupings of cortical regions derived from the Draw-EM pipeline. The key reasons for training an extra network are: first, we avoid the time consuming task of label propagation between our available dHCP Draw-EM output segmentations and predicted ePrime maps, and second, we can train this network using Draw-EM cortical segmentations, and apply it on any brain cortical gray matter maps as in this case there will be no intensity shift between target and source distributions.

**Table 2:** Grouping of cortical substructures showing their original tissue name obtained from Draw-EM (Makropoulos et al., 2018) on the first column and their corresponding cortical subregion on the second column.

**Tissue name**	**Cortical subregion**
Anterior temporal lobe, medial part left	
Anterior temporal lobe, lateral part left	
Gyri parahippocampalis et ambiens anterior part left	
Superior temporal gyrus, middle part left	
Medial and inferior temporal gyri anterior part left	
Lateral occipitotemporal gyrus, gyrus fusiformis anterior part left	Temporal (left)
Gyri parahippocampalis et ambiens posterior part left	
Lateral occipitotemporal gyrus, gyrus fusiformis posterior part left	
Medial and inferior temporal gyri posterior part left	
Superior temporal gyrus, posterior part left	
Anterior temporal lobe, medial part right	
Anterior temporal lobe, lateral part right	
Gyri parahippocampalis et ambiens anterior part right	
Superior temporal gyrus, middle part right	
Medial and inferior temporal gyri anterior part right	
Lateral occipitotemporal gyrus, gyrus fusiformis anterior part right	Temporal (right)
Gyri parahippocampalis et ambiens posterior part right	
Lateral occipitotemporal gyrus, gyrus fusiformis posterior part right	
Medial and inferior temporal gyri posterior part right	
Superior temporal gyrus, posterior part right	
Insula left	Insula (left)
Insula right	Insula (right)
Occipital lobe left	Occipital (left)
Occipital lobe right	Occipital (right)
Cingulate gyrus, anterior part right	
Cingulate gyrus, anterior part left	Cingulate
Cingulate gyrus, posterior part right	
Cingulate gyrus, posterior part left	
Frontal lobe left	Frontal (left)
Frontal lobe right	Frontal (right)
Parietal lobe left	Parietal (left)
Parietal lobe right	Parietal (right)

### 2.3. Network Architectures

The segmentation networks in all three setups and the generator used in the adversarial domain adaptation in the image space model have the same architecture, consisting of 5 encoding-decoding branches with 16, 32, 64, 128, and 256 channels, respectively. The encoder blocks use 3^3^ convolutions (with a stride of 1), instance normalization (Ulyanov et al., 2016) and LeakyReLU activations. A 2^3^ average pooling layer is used after the first down-sampling block, while the others use 2^3^ max pooling layers. The decoder blocks consist of 3^3^ convolutions (with a stride of 1), instance normalization (Ulyanov et al., 2016), LeakyReLU activations, and, additionally, 3^3^ transposed convolutions. The number of encoding-decoding blocks, as well as the use of LeakyReLU activations and instance normalization layers, were chosen based on the best practices described in Isensee et al. (2018). At the same time, the network configurations that we have chosen allowed us to work with the hardware we have at hand (Titan XP 12 GB). The segmentation network outputs a 7-channel 3D volume (of the same size as the input image), corresponding to our 7 classes: background, cerebrospinal fluid (CSF), cortical gray matter (cGM), white matter (WM), deep gray matter (dGM), cerebellum and brainstem. The generator network's last convolutional layer is followed by a Tanh activation and outputs a single channel image.

For our unsupervised domain adaptation models (Figures 3, 4) we used a PatchGAN discriminator as proposed in Isola et al. (2017). Its architecture consists of 5 blocks of 4^3^ convolutions (with a stride of 2) with 64, 128, 256, 512, and 1 channels, respectively), instance normalization and LeakyReLU activations.

The cortical parcellation network has the same architecture as the tissue segmentation network, but outputs a 12-channel 3D volume corresponding to the following cortical substructures: frontal left, frontal right, cingulate, temporal left, temporal right, insula left, insula right, parietal left, parietal right, occipital left, and occipital right, respectively. The last class represents the background.

### 2.4. Training

The baseline segmentation network (Figure 2) was trained by minimizing the generalized Dice loss (Sudre et al., 2017) between the predicted and the Draw-EM segmentation maps (Equation 1).

(1) $$\begin{array}{r} L_{method_{1}}=L_{seg}=1-2\frac{\sum_{l=1}^{M}w_{l}\sum_{n}p_{ln}t_{ln}}{\sum_{l=1}^{M}w_{l}\sum_{n}p_{ln}+t_{ln}} \end{array}$$

where $w_{l}=1/{\left(\underset{n}{\sum }t_{ln}\right)}^{2}$ is the weight of the lth tissue type, *p*_*ln*_ is the predicted probabilistic map of the lth tissue type at voxel n, *t*_*ln*_ is the target label map of the lth tissue type at voxel n, and M is the number of tissue classes. While training, we used the Adam optimizer (Kingma and Ba, 2014) with its default parameters and a decaying cyclical learning rate scheduler (Smith, 2017) with a base learning rate of 2·10^−6^ and a maximum learning rate of 2·10^−3^. The choice of optimizer was based on knowledge of previous image translation literature (Isola et al., 2017; Zhu et al., 2017; Liao et al., 2019; Ranzini et al., 2020) where it yielded good results. At the same time, a varying learning rate during training was shown to improve results in fewer iterations when compared to using a fixed value (Smith, 2017).

The segmentation network from the adversarial domain adaptation in the latent space model was trained to produce tissue maps on the source (dHCP) volumes. In addition, both target (ePrime) and source (dHCP) volumes were fed to the segmentation network, while the feature maps obtained from every level of its decoder arm were passed to the discriminator network which acted as a domain classifier. This was done after either up-sampling or down-sampling the feature maps to match the volume size of the second deepest layer. This model was trained by minimizing a Cross-Entropy loss between predicted and assigned target labels representing our two domains. The final loss function for our second model was therefore made up of the generalized Dice loss and an adversarial loss:

(2) $$\begin{array}{r} L_{method_{2}}=L_{seg}-\alpha L_{adv} \end{array}$$

where *α* was a hyperparameter increased linearly from 0 to 0.05 starting at epoch 20, and which remained equal to 0.05 from epoch 50 onward. Similar to Kamnitsas et al. (2017) we looked at the behavior of our discriminator and segmentation network when training with different values of *α* ∈ [0.02, 0.05, 0.1, 0.2, 0.5]. We found the discriminator's accuracy during training stable for all investigated values, while the segmentation network achieved the lowest loss when *α* = 0.05. The segmentation network was trained similarly to the baseline model, while the discriminator network was trained using the Adam optimizer with *β*_1_ = 0.5 and *β*_2_ = 0.999, and a linearly decaying learning rate scheduler starting from 2·10^−3^.

The generator network used in the image space domain adaptation approach was trained to produce synthesized ePrime volumes, while the segmentation network was trained using the same loss function, optimizer and learning rate scheduler as in the other two methods. In the previous model (adversarial domain adaptation in the latent space) we fed both dHCP and ePrime volumes to the segmentation network to obtain data agnostic feature maps. For this reason, and to allow for a fair comparison between the two unsupervised domain adaptation models, we trained the segmentation network from the image space model on both real dHCP and synthesized ePrime volumes. For both the discriminator and the generator networks the Adam optimizer with *β*_1_ = 0.5 and *β*_2_ = 0.999 was used, together with a linearly decaying learning rate scheduler starting from 2·10^−3^. The loss function of the discriminator was similar to that of the Least Squares GAN (Mao et al., 2017): $L_{D}=E_{x\sim T}\left[{\left(D\left(x\right)-b\right)}^{2}\right]+E_{x\sim S}\left[{\left(D\left(G\left(x\right)\right)-a\right)}^{2}\right]$ where *a* signified the label for synthesized volumes and *b* was the label for real volumes. The generator and the segmentation network were trained together using the following loss:

(3) $$\begin{array}{r} L_{method_{3}}=L_{seg}+L_{adv} \end{array}$$

where $L_{adv}=E_{x\sim S}\left[{\left(D\left(G\left(x\right)\right)-b\right)}^{2}\right]$. An additional NCC loss was used between the real and the generated volumes in order to constrain the generator to produce realistic looking ePrime-like images. Without the additional NCC loss, the generator tends to produce images with an enlarged CSF boundary in order to match the preterm-only distribution found in the ePrime dataset, as was previously shown in Grigorescu et al. (2020).

These three methods were trained with and without data augmentation for 100 epochs, during which we used the validation sets to inform us about our models' performance and to decide on the best performing models. For data augmentation we applied: random affine transformations [with rotation angles $\theta_{i}\sim U\left(-10^{\text{o}},10^{\text{o}}\right)$ and/or scaling values $s_{i}\sim U\left(0.8,1.2\right)$], random motion artifacts [corresponding to rotations of $\theta_{i}\sim U\left(-2^{\text{o}},2^{\text{o}}\right)$ and translations of $t_{i}\sim U\left(-2\text{mm},2\text{mm}\right)$], and random MRI spike and bias field artifacts (Pérez-García et al., 2020). The cortical parcellation network was trained in a similar fashion as the baseline tissue segmentation network, with data augmentation in the form of random affine transformations (with the same parameters as above).

The test set was used to report our final models' results and to showcase their capability to generalize on the source domain. Finally, we produced tissue segmentation maps for all the subjects in our datasets, and used them as input into ANT's DiReCT algorithm (Tustison et al., 2013) to compute cortical thickness measures. To validate our results, we compared cortical thickness measures between subsets of the two cohorts matched for GA and PMA, for which we expect no significant difference in cortical thickness if the harmonization was successful. We also assessed the association between PMA and cortical thickness in the two cohorts.

## 3. Results

### 3.1. dHCP Test Dataset

#### 3.1.1. Baseline and Domain Adaptation Models

**Figure 5:**
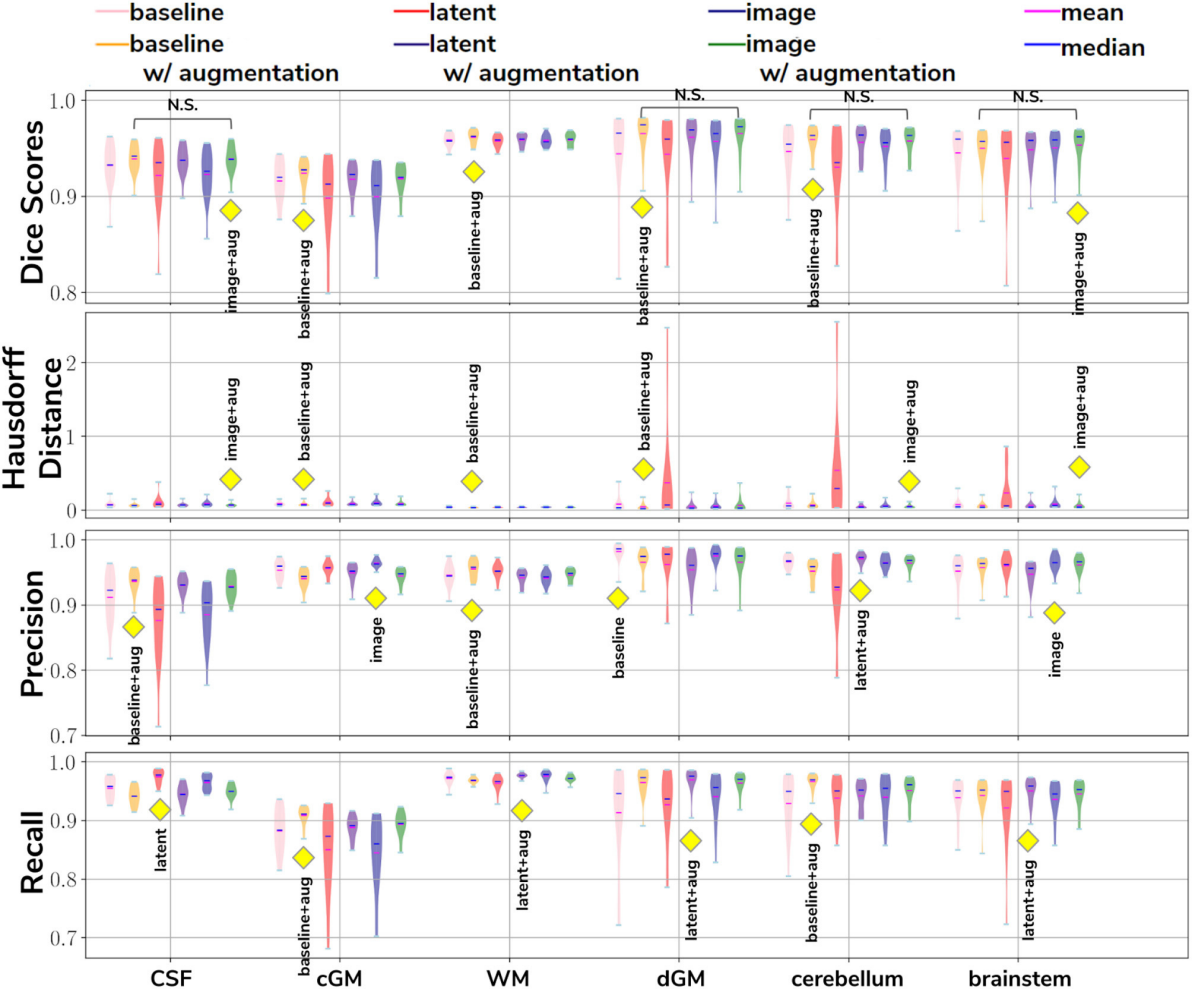
The results on our dHCP test dataset for all six methods. The yellow diamond highlights the model which obtained the best mean score for its respective tissue type and metric. Models which obtained non-significant differences when compared to the best performing method are shown above each pair.

In our first experiment we looked at the performance of the six trained models when applied to the source (dHCP) test dataset. The aim was to assess whether our trained models were able to generalize to unseen source domain (dHCP) data for which we have reliable Draw-EM outputs. Figure 5 summarizes the results of our trained models, showing mean Dice scores, mean Hausdorff distance calculated using SimpleITK (Lowekamp et al., 2013; Yaniv et al., 2018), precision and recall. These metrics were computed between the predicted tissue segmentation maps and the Draw-EM output labels for each of the six trained models. The model that obtained the best score is highlighted with the yellow diamond for each metric and tissue type. In terms of Dice scores, out of the six models, the *baseline with augmentation* and *image with augmentation* methods performed best on the source domain test dataset for CSF, dGM, cerebellum and brainstem, with no significant difference between them. For cGM and WM, the best performance was obtained by the *baseline with augmentation* model, while the domain adaptation methods showed a slight decrease in performance. The three models trained without augmentation always performed significantly worse than their augmented counterparts.

In terms of average Hausdorff distance, both the *baseline with augmentation* and *image with augmentation* models performed well, while the *latent without augmentation* model performed worse than all the other models for all tissue types. Highest precision scores were obtained by the *baseline with augmentation* model for both CSF and WM, the *image without augmentation* method for both cGM and brainstem, the *baseline without augmentation* for dGM, and the *latent with augmentation* model for cerebellum. Highest recall scores were obtained by the *baseline with augmentation* model for cGM and cerebellum, the *latent with augmentation* model for WM, dGM and brainstem, and the *latent without augmentation* model for CSF. These results show that our trained models were able to generalize to unseen source domain data, and that the performance on the dHCP dataset was not compromised by using domain adaption techniques.

#### 3.1.2. Cortical Parcellation Network

To assess the performance of our trained cortical parcellation network, we applied it on the source (dHCP) test dataset, where the inputs were binary Draw-EM cortical gray matter tissue maps. For each subject in our test dataset, the network produced a 12-channel output, consisting of: frontal left, frontal right, cingulate, temporal left, temporal right, insula left, insula right, parietal left, parietal right, occipital left, occipital right, and background, respectively. Table 3 summarizes these results in terms of minimum, maximum and mean Dice scores for each of the 11 cortical substructures. When compared with the Draw-EM outputs (Makropoulos et al., 2018), the network obtained an overall mean Dice score of 0.97.

**Table 3:** Dice Scores obtained on the dHCP test set for the trained cortical parcellation network.

**Tissue**	**Min**	**Max**	**Mean**	**Tissue**	**Min**	**Max**	**Mean**
Frontal (left)	0.98	0.99	0.99	Frontal (right)	0.98	0.99	0.99
Temporal (left)	0.96	0.99	0.98	Temporal (right)	0.97	0.98	0.98
Insula (left)	0.95	0.97	0.96	Insula (right)	0.95	0.97	0.96
Parietal (left)	0.96	0.98	0.97	Parietal (right)	0.96	0.98	0.97
Occipital (left)	0.94	0.98	0.97	Occipital (right)	0.95	0.98	0.97
Cingulate	0.93	0.97	0.96

### 3.2. Validation of Data Harmonization

In order to evaluate the extent to which each of the trained models managed to harmonize the segmentation maps of the two cohorts, we looked at tissue volumes and mean cortical thickness measures between subsamples of the dHCP (*N* = 30; median GA = 30.50 weeks; median PMA = 41.29 weeks) and ePrime (*N* = 30; median GA = 30.64 weeks; median PMA = 41.29 weeks) cohort which showed comparable GA at birth and PMA at time of scan (see Table 1). A direct comparison between the two cohort subsets shows that the dHCP and ePrime neonates did not differ significantly in terms of sex [χ^2^(1) < 0.001, *p* > 0.05], or maternal ethnicity [χ^2^(4) = 4.32, *p* > 0.05], coded as “white or white British,” “black or black British,” “asian or asian British,” “mixed race,” and “other.” As a proxy for socio-economic status, we derived an Index of Multiple Deprivation (IMD) score based on parental postcode at the time of infant birth (Department for Communities and Local Government, 2011^3^). This measure is based on seven domains of deprivation within each neighborhood compared to all others in the country: income, employment, education, skills and training, health and disability, barriers to housing and services, living environment and crime. Higher IMD values therefore indicate higher deprivation. IMD score did not differ significantly between dHCP (*M* = 21.4, *SD* = 10.7) and ePrime (*M* = 18.0, *SD* = 11.6) subsets, suggesting that these two groups are comparable in terms of environmental background.

For these two cohort subsamples with similar GA and PMA, we expected both volumes and cortical thickness measures not to differ after applying the harmonization procedures. We also investigated the relationship between PMA and volumes and cortical thickness respectively, before and after applying the harmonization. Linear regressions were performed in the comparable data subsets testing the effects of PMA and cohort on volumes (or cortical thickness), controlling for GA and sex.

#### 3.2.1. Volumes

**Figure 6:**
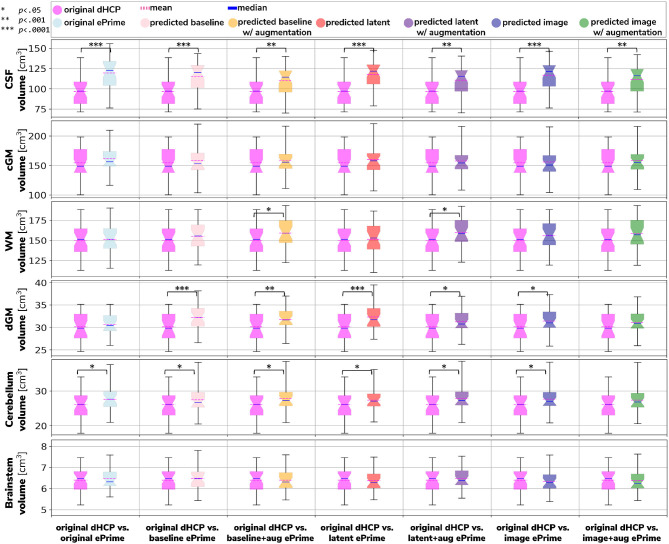
Comparison of volume measures for our six tissue types (CSF, cGM, WM, dGM, cerebellum, and brainstem) between original Draw-EM dHCP segmentations and original Draw-EM ePrime segmentations (first column), or between original Draw-EM dHCP segmentations and ePrime segmentations obtained with the six trained models (columns 2–7). Linear regressions were performed in the comparable data subsets testing the effects of cohort on volumes, controlling for PMA, GA, and sex (volume ~ cohort + PMA + GA + sex). The asterisks indicate a statistically significant effect of cohort in the linear regression.

Figure 6 shows the tissue volumes for both the original and the predicted segmentations. Significant volume differences between the two subsamples (i.e., significant effect of cohort in the regression model) are reported above each tested model. To summarize, the *image with augmentation* model performed best, by showing no significant differences in the two cohorts for cortical gray matter, white matter, deep gray matter, cerebellum and brainstem. The cerebrospinal fluid volumes were significantly different between the two cohorts for all our trained models, as well as for the original ePrime segmentation masks.

#### 3.2.2. Cortical Thickness

**Figure 7:**
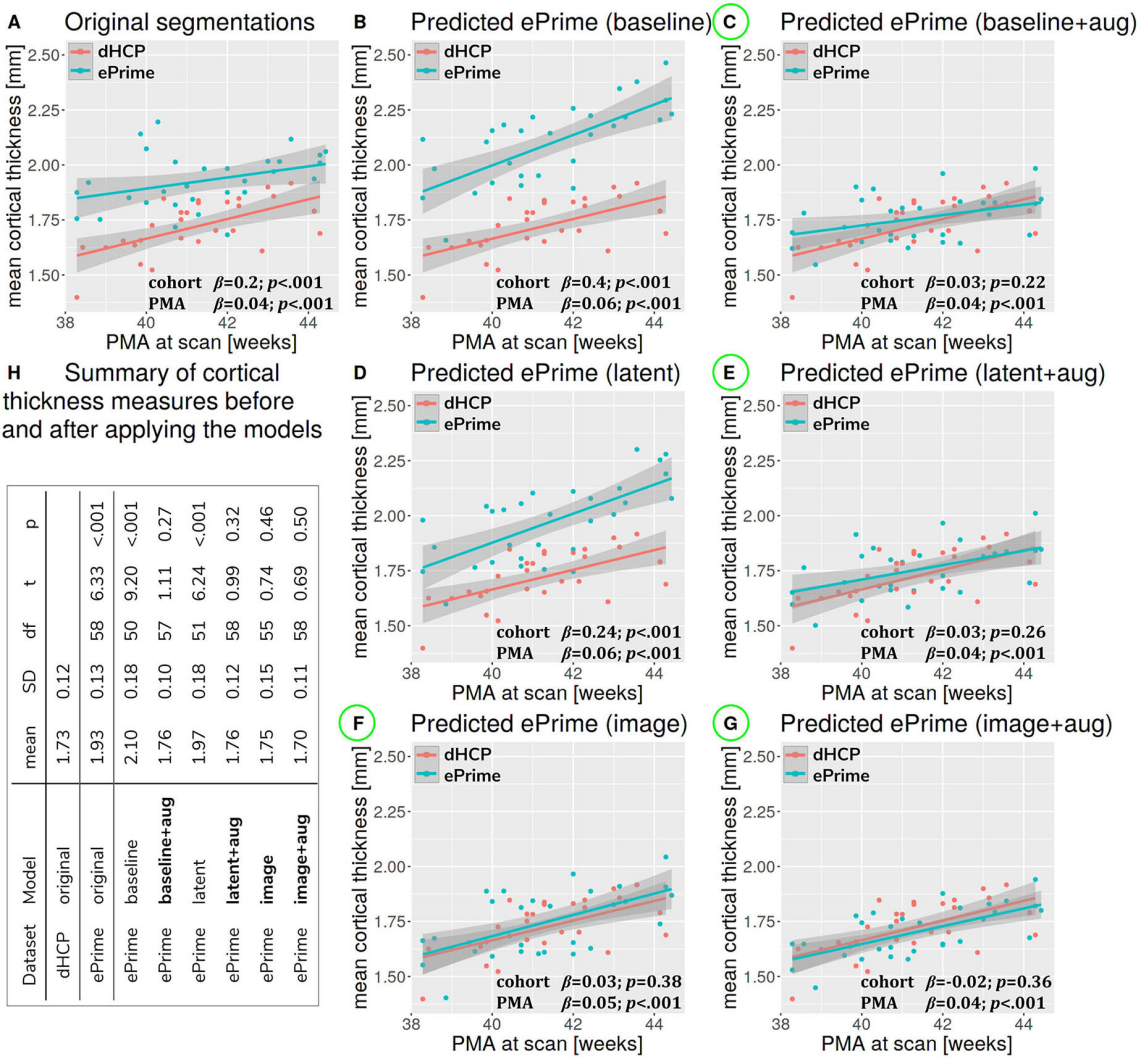
The association between PMA and mean cortical thickness before **(A)** and after **(B–G)** applying the data harmonization models on the matched dHCP and ePrime subsets. A linear model regressing mean cortical thickness measures on PMA, GA, sex, and cohort revealed a significant effect of cohort for the original segmentations **(A)**, and the predicted maps (**B** - *baseline without augmentation* and **D** - *latent without augmentation*). The effect of cohort was rendered non-significant for four of the methods (**C** - *baseline with augmentation*, **E** - *latent with augmentation*, **F** - *image without augmentation*, and **G** - *image with augmentation*). **(H)** summarizes cortical thickness measures before and after applying the models.

Figure 7 summarizes the results of applying the cortical thickness algorithm on the predicted segmentation maps for all six methods. Before harmonization, the matched subsets from the dHCP and ePrime cohorts showed a significant difference in mean cortical thickness [dHCP: *M* = 1.73, *SD* = 0.12; ePrime: *M* = 1.93, *SD* = 0.13; *t*(58) = 6.33, *p* < 0.001]. After applying the harmonization to the ePrime sample, mean cortical thickness no longer differed between the two subsamples for four of our methods. These results are summarized in panel H from Figure 7, where the models which obtained harmonized values in terms of mean cortical thickness measures are shown in bold. Figure 7 also shows the association between PMA and mean crtical thickness before (Figure 7A) and after applying the models (Figures 7B–G) on the matched dHCP and ePrime subsets. A linear model regressing unharmonized mean cortical thickness on PMA, GA, sex, and cohort revealed a significant effect of cohort (*β* = 0.20; *p* < 0.001), consistent with a group difference in mean cortical thickness reported above, as well as a significant effect of PMA (*β* = 0.04; *p* < 0.001), consistent with an increase in cortical thickness with increasing PMA. After applying the methods, the effect of cohort was rendered non-significant for four of the methods (see highlighted panels C, E, F, G from Figure 7), while the effect of PMA remained stable across all six methods.

**Figure 8:**
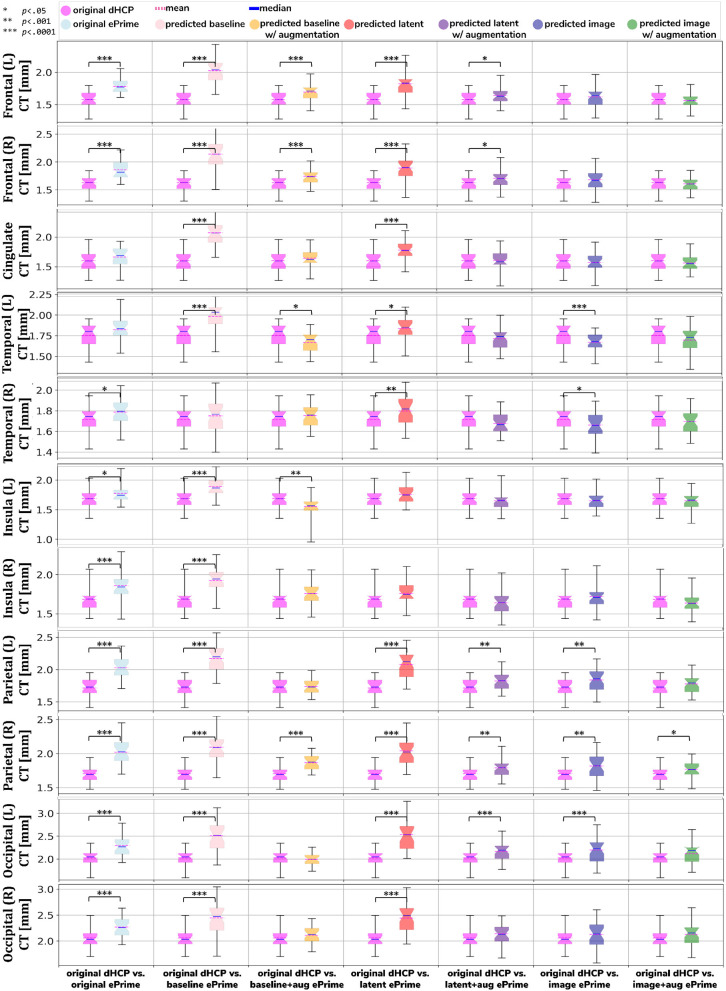
Comparison of local mean cortical thickness measures between original Draw-EM dHCP segmentations and original Draw-EM ePrime segmentations (first column), or between original Draw-EM dHCP segmentations and ePrime segmentations obtained with the six trained models (columns 2–7). Linear regressions were performed in the comparable data subsets testing the effects of cohort on local cortical thickness measures, controlling for PMA, GA, and sex (CT ~ cohort + PMA + GA + sex). The asterisks indicate a statistically significant effect of cohort in the linear regression.

We performed a similar analysis on thickness measures of the cortical substructures. To obtain these measures, we used the original and the predicted cortical gray matter segmentation maps (obtained by applying each of our six methods) as input to the trained cortical parcellation network to predict cortical substructure masks. We then used these masks to calculate local cortical thickness measures. Our results are summarized in Figure 8.

#### 3.2.3. Example Predictions

**Figure 9:**
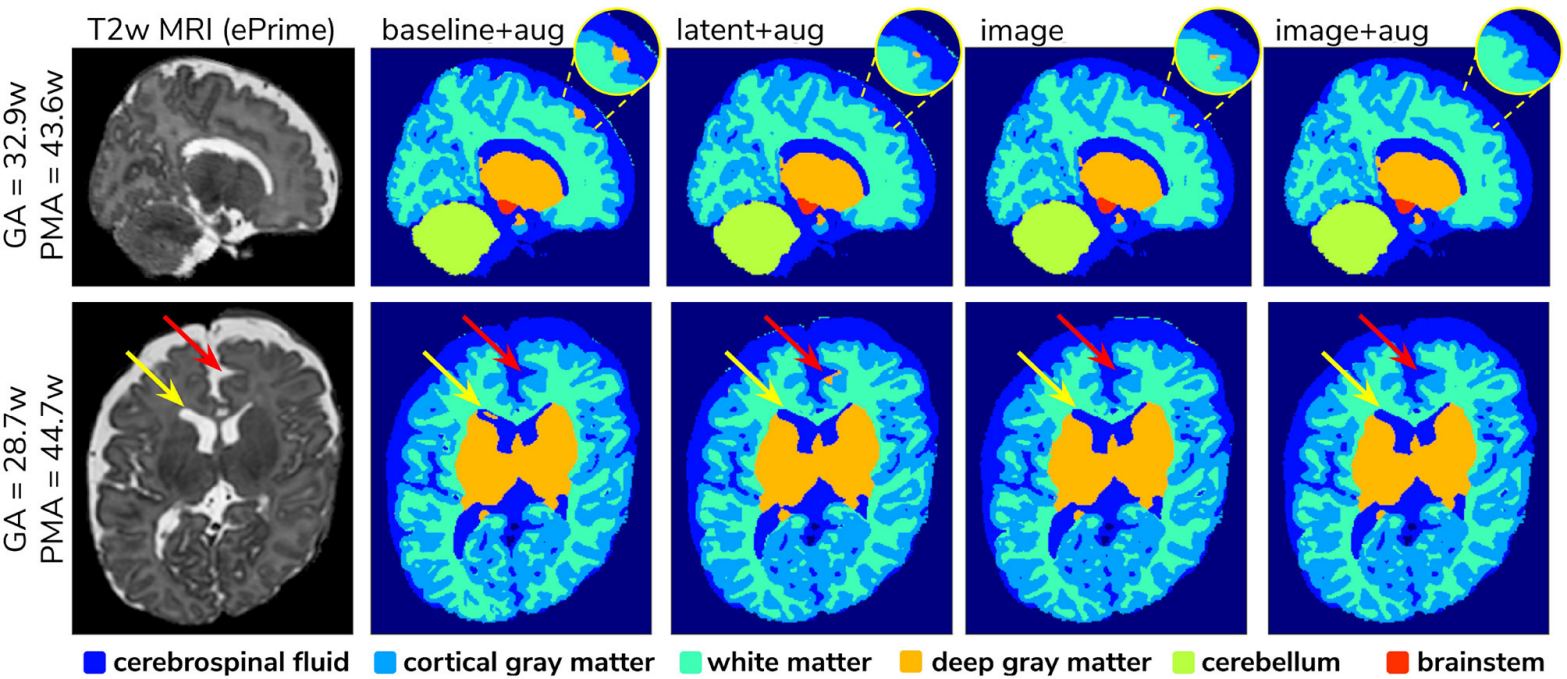
Example predicted segmentation maps for the best performing models. On the first row we show an example where three of the models (*baseline with augmentation, latent with augmentation*, and *image*) misclassified a part of the cortex as being deep gray matter. This is more pronounced in the *baseline with augmentation* model, while the *latent with augmentation* and *image* show a slight improvement. The *image with augmentation* model corrected the problem entirely. On the second row the yellow arrow points to an area of CSF where the *baseline with augmentation* model misclassified it as dGM, while the other three models did not have this problem. The red arrow on the other hand points to an area where the *latent with augmentation* model misclassified cGM as deep gray matter. This problem does not appear in the other models.

To further narrow down which of the four remaining methods was best at harmonizing our ePrime neonatal dataset, we looked at the predicted segmentations. Figure 9 shows two example neonates from the ePrime dataset with GA = 32.9 w, PMA = 43.6 w, and with GA = 28.7 w, PMA = 44.7 w, respectively. The first column shows *T*2w saggittal and axial slices, respectively, while the following four columns show example tissue prediction maps produced by the four models: *baseline with augmentation, latent with augmentation, image*, and *image with augmentation*, respectively. Although all four methods performed well in terms of harmonizing tissue segmentation volumes and global mean cortical thickness values for the two subsamples with similar GA and PMA, previously presented quantitative results as well as the example above suggest that the *image with augmentation* method was more robust.

**Figure 10:**
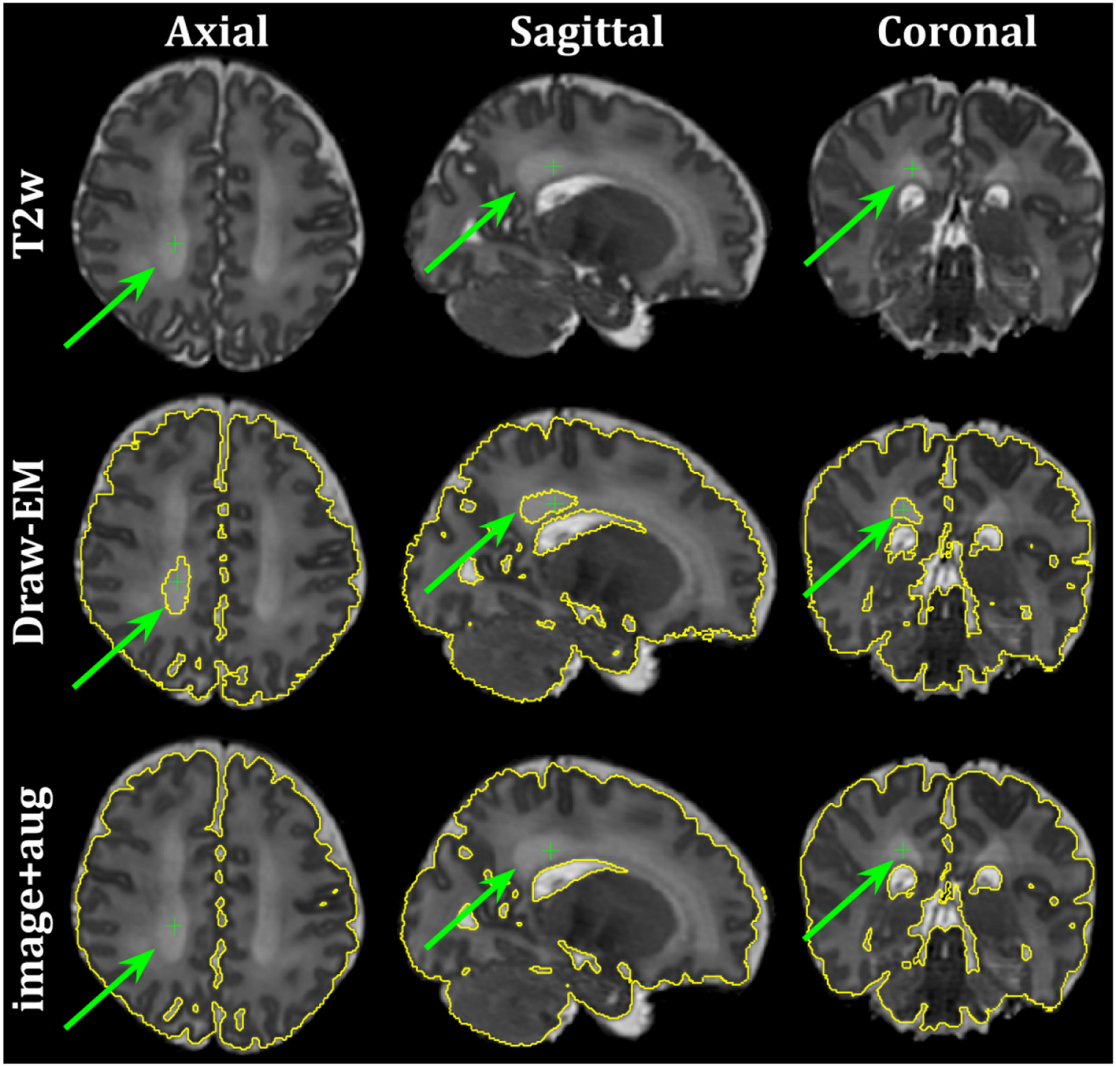
Example of a neonate from the ePrime dataset with GA = 32.86 w and PMA = 39.86 w where the Draw-EM algorithm performed worse than our proposed *image with augmentation* model. The green arrow points at a region which was segmented as CSF by Draw-EM, but then corrected by our model.

Finally, Figure 10 shows the axial, sagittal and coronal slices of an ePrime neonate (GA = 32.86 w and PMA = 39.86 w). The first line shows the *T*2w MR image, while the second and third lines show the CSF boundary of both the Draw-EM algorithm and the *image with augmentation* method. The green arrows point to a WM region which was misclassified by the Draw-EM pipeline as CSF. This problem was then corrected by the *image with augmentation* method.

### 3.3. Analysis of Harmonized Cortical Substructures

In this section we analyze the harmonized cortical gray matter segmentation maps using the *image with augmentation* model. We produce tissue segmentation maps for the entire ePrime dataset and calculate cortical thickness measures on the predicted and Draw-EM cortical gray matter tissue maps of both cohorts. In addition, we use the trained cortical parcellation network to produce cortical substructure masks. We perform a term *vs* preterm analysis on the harmonized cortical gray matter maps and we show the importance of harmonizing the data with a proof-of-principle application setting where we investigate the association between cortical thickness and a language outcome measure.

#### 3.3.1. Comparison of Term and Preterm Cortical Maps

**Figure 11:**
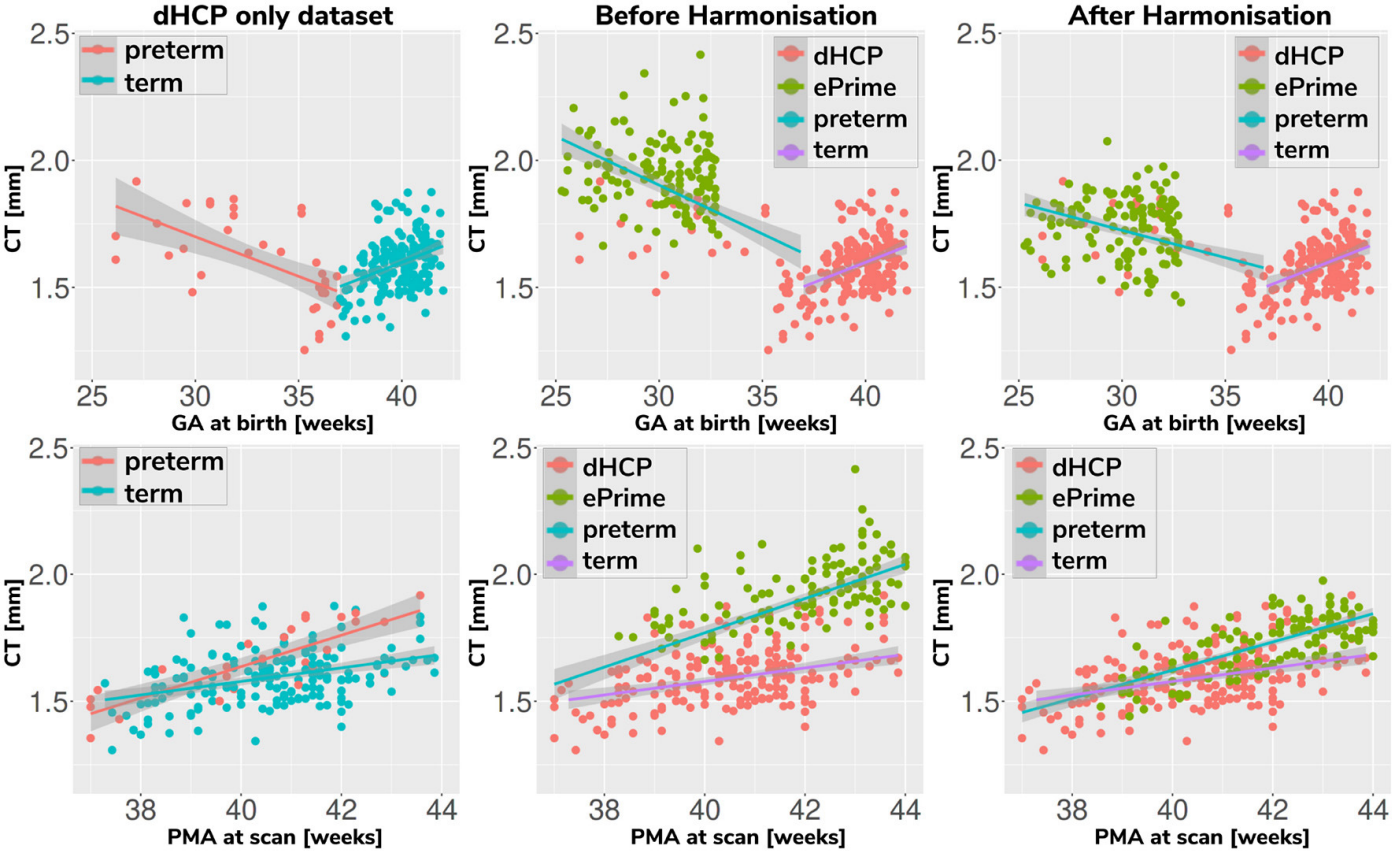
Mean cortical thickness measures in our dHCP dataset (first column), and in both cohorts before (second column) and after (third column) harmonizing the tissue segmentation maps. The first row plots the cortical thickness measures against GA, while the second row plots the cortical thickness measures against PMA, with individual regression lines on top.

Associations between cortical thickness and GA or PMA in the full dHCP and ePrime datasets (excluding subjects with PMA >45 weeks) for the whole cortex are depicted in Figure 11, where we show individual regression lines for preterm-born and term-born neonates. The first column consists of dHCP-only subjects, while the following two columns showcase both cohorts together, before and after harmonizing the cortical gray matter tissue maps.

A linear model regressing dHCP-only mean cortical thickness on PMA, GA, sex, birth weight and the interaction between PMA and GA revealed a significant effect of PMA (*β* = 0.19; *p* < 0.001), a significant effect of GA (*β* = 0.16; *p* = 0.002), and a significant effect of the interaction between PMA and GA (*β* = −0.004; *p* = 0.002), indicating that infants born at a lower GA showed a stronger relationship between PMA and CT. When performing the same analysis in the pooled ePrime and dHCP data before harmonizing the maps, the effect of GA and the effect of the interaction were rendered not significant (GA: *β* = 0.009; *p* = 0.7 and PMA*GA: *β* = −0.0006; *p* = 0.5, respectively). This is corrected after harmonizing the tissue maps, where the effects of GA (*β* = 0.06; *p* = 0.02) and the effects of the GA and PMA interaction (*β* = −0.001; *p* = 0.02) are, again, significant.

The second and third columns of Figure 11 show that after harmonizing the tissue segmentation maps, the ePrime preterm-born neonates (green dots) are brought downwards into a comparable range of values to the dHCP preterms (red dots). Moreover, when plotting the cortical thickness measures against PMA, after harmonizing the tissue maps, the intersection between the two individual regression lines (term and preterm-born neonates) happens at roughly the same age (PMA = 38.5 weeks) as in the dHCP-only dataset.

**Figure 12:**
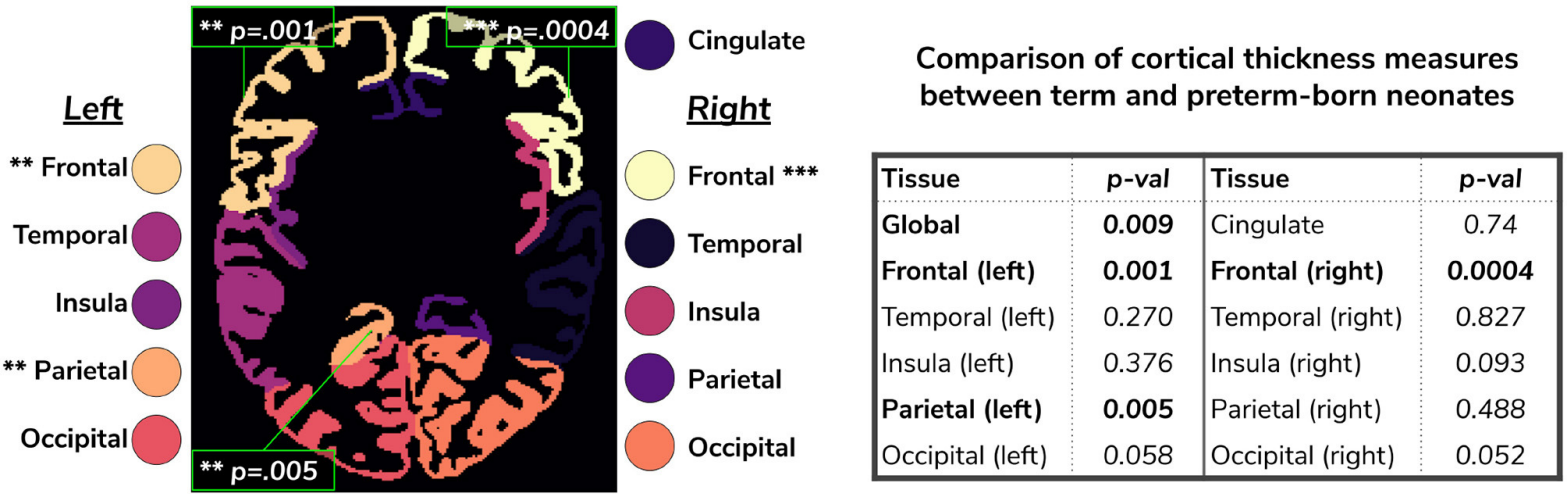
Comparison of cortical thickness measures for the whole cortex and for each of the 11 cortical subregions between term and preterm-born neonates. The results of the linear regression are reported in the table in terms of differences between term and preterm-born neonates.

We extended the term *vs* preterm analysis on cortical thickness substructures. Figure 12 shows the results of applying a linear model regressing mean cortical thickness measures on PMA, GA, sex, birth weight and prematurity, where significant differences (*p* < 0.05) between the two cohorts (term and preterm-born neonates) are highlighted in the image.

#### 3.3.2. Behavioral Outcome Association

As a final proof-of-principle, we demonstrate the importance of data harmonization in an application setting investigating the association between neonatal cortical thickness and a behavioral outcome measure. For this, we consider language abilities as assessed between 18 and 24 months in both dHCP and ePrime cohorts using the Bayley Scales of Infant and Toddler Development (Bayley, 2006). Age-normed composite language scores were available for 203 toddlers from the dHCP cohort (M = 96.43; SD = 14.89) and 136 toddlers from the ePrime cohort (M = 91.25; SD = 17.37). For the neonatal cortical thickness measure, we focus on the left and right frontal cortex for illustration.

Regressing composite language score against left or right frontal cortical thickness in each cohort separately, controlling for PMA, GA, sex and intracranial volume showed that there was no significant association between neonatal left/right frontal cortical thickness and language abilities at toddler age in either of the cohorts. However, when pooling data from both cohorts together and rerunning the same analysis (using un-harmonized cortical thickness measures), a significant association between left/right frontal cortical thickness and language abilities is seen (left: *β* = −17.56, *p* < 0.05, right: *β* = −18.76, *p* < 0.05), suggesting that greater frontal cortical thickness at term-equivalent age is associated with reduced language abilities at toddler age.

**Figure 13:**
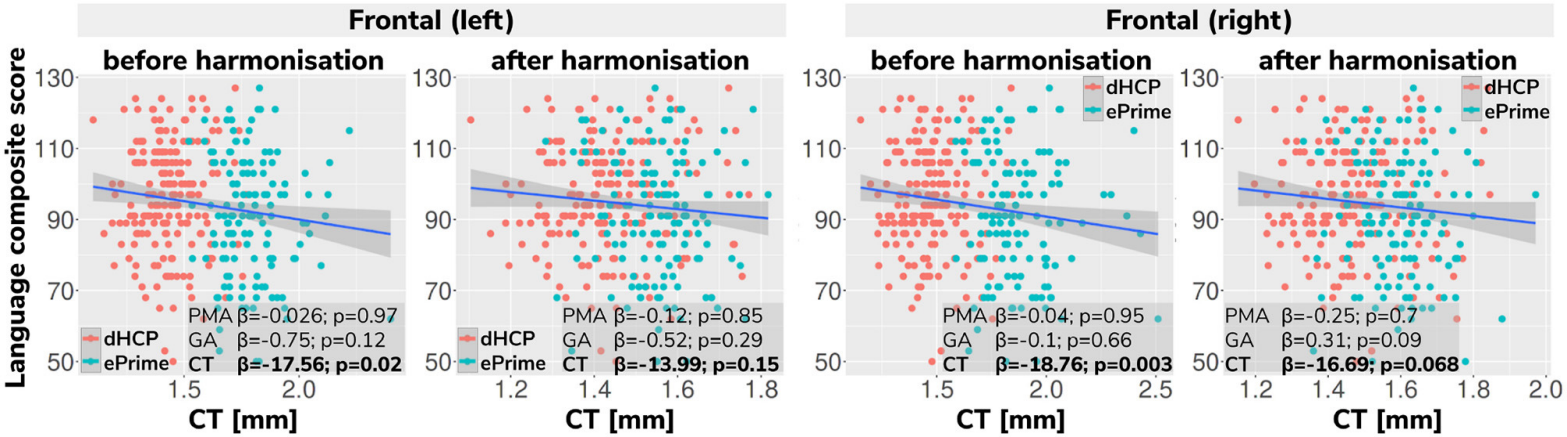
Language composite score against predicted left and right frontal cortical thickness measures before and after harmonizing the tissue segmentation maps. Without harmonization (columns 1 and 3) there appears to be a significant association between left or right frontal cortical thickness and language abilities, but after harmonization (columns 2 and 4) the effect of cortical thickness on language ability is rendered non-significant in both left and right frontal cortex. This demonstrates the importance of data harmonization without which pooling images from separate datasets can lead to spurious findings that are driven by differences in acquisitions rather than by true underlying effects.

However, as can be seen in Figure 13, this is likely a spurious effect due to (artifactually) heightened cortical thickness values in un-harmonized ePrime data combined with lower language composite scores in the ePrime cohort (consistent with effects typically observed in preterm cohorts). Indeed, when rerunning the same analysis on harmonized data pooled across both cohorts, the effect of cortical thickness on language ability is rendered non-significant in both left (*β* = −13.99, *p* = 0.15) and right (*β* = −16.69, *p* = 0.068) frontal cortex, consistent with the ground-truth findings in each individual cohort.

## 4. Discussion and Future Work

In this paper we studied the application and viability of unsupervised domain adaptation methods for harmonizing tissue segmentation maps of two neonatal datasets (dHCP and ePrime). Our aim was to obtain volumetric and cortical thickness measures that are only affected by brain anatomy and not by the acquisition protocol or scanner, in order to improve the statistical power of imaging or imaging-genetic studies. We proposed an image-based domain adaptation model where a tissue segmentation network was trained with real dHCP and synthesized ePrime *T*2w 3D MRI volumes. The generator network was trained to produce realistic images in order to fool a domain discriminator, while also minimizing an NCC loss which aimed to enforce image similarity between real and synthesized images (Grigorescu et al., 2020). We trained this model using dHCP Draw-EM segmentation maps, and we compared it with a baseline 3D U-Net (Çiçek et al., 2016), and a latent space domain adaptation method (Kamnitsas et al., 2017). The three methods were trained with and without data augmentation (Pérez-García et al., 2020).

First, we looked at the performance of each of the six trained models on the source (dHCP) test dataset, by comparing predicted tissue segmentation maps with the Draw-EM output labels, with the aim of measuring fidelity of our trained segmentation methods for the original dHCP domain. Our results on the source (dHCP) test dataset suggest that our trained models were able to generalize to unseen source domain data. At the same time, Dice score results on the test set for the proposed *image with augmentation* model are high and are similar in performance when compared with the *baseline with augmentation* method. This suggests that adding the contrast transfer step does not diminish the quality of the segmentations.

We then analyzed the extent to which each of the 6 trained models managed to harmonize the tissue segmentation maps of our two cohorts, by looking at tissue volumes and mean cortical thickness measures between subsamples of the dHCP and ePrime cohorts which showed comparable GA at birth and PMA at time of scan, as well as similar gender and maternal ethnicity. Our results showed that our proposed model (*image with augmentation*) harmonized the predicted tissue segmentation maps in terms of cortical gray matter, white matter, deep gray matter, cerebellum and brainstem volumes (Figure 6). In terms of mean global cortical thickness measures, four of the trained methods (*baseline with augmentation, latent with augmentation, image*, and *image with augmentation*) achieved comparable values when compared to the dHCP subset. In fact, we hypothesize that these four methods provided the best overall results because either they were trained using data augmentation or they acted as a deep learning-based augmentation technique (Sandfort et al., 2019), which made the segmentation network more robust to the different contrast, population bias and acquisition protocol of the ePrime dataset.

Using the cortical parcellation network, we also produced cortical thickness measures for the 11 cortical subregions (see Table 2). Again, the models trained with augmentation performed better than their no augmentation counterparts (see Figure 8). However, our proposed *image with augmentation* model performed best, whereby ePrime values, tending toward higher values before harmonization, were brought downwards into a comparable range of values to dHCP, for 10 out of 11 cortical subregions (see Figure 8 last column). For the right parietal lobe, our proposed method outperformed the original segmentations and the other 5 models, but did not manage to bring the values down to a non-significant range. One potential reason for this is that, on a visual inspection, the ePrime cohort appears to suffer from more partial volume artifacts than its dHCP counterpart, which can confuse the segmentation network and can lead to overestimation of the cortical gray matter/cerebrospinal fluid boundary.

A close inspection of the predicted tissue segmentation maps (see Figure 9) also showed that our proposed model (*image with augmentation*) corrected misclassified voxels which were prevalent in the other 3 methods. At the same time, the proposed *image with augmentation* method outperformed the original Draw-EM segmentation by correcting a region of WM which was wrongly classified as CSF (see Figure 10). Our results suggest that, in terms of consistency of volumes and regional cortical thickness measures derived from dHCP and ePrime neonates (Figures 6, 8), as well as the qualitative examples (Figures 9, 10), our proposed *image with augmentation* model resulted in more consistent outputs than the other methods.

We used the harmonized cortical segmentation maps to look at differences in both global and local cortical thickness measures between term and preterm-born neonates. We showed in Figure 12 that our harmonized cortical gray matter maps resulted in global thickness measures which were comparable with the dHCP-only neonates, while also revealing a significant effect of GA and the interaction between age at scan and at birth. We performed a similar analysis on the local cortical thickness measures and highlighted three regions of interest (frontal left, frontal right, and parietal left) which showed significant differences between the two cohorts (see Figure 12). These regions are consistent with previous studies (Nagy et al., 2011) where cortical thickness measures were shown to differ in preterm-born neonates when compared to term-born neonates in an adolescent cohort.

Finally, we showed the importance of harmonizing the cortical tissue maps by investigating the association between neonatal cortical thickness and a language outcome measure. After harmonization, regressing language composite score against predicted left or right frontal cortical thickness in the two pooled datasets, showed no significant effect of cortical thickness (second column of Figure 13), consistent with the ground-truth results seen in each cohort individually. This analysis demonstrates that without data harmonization, pooling images from separate datasets can lead to spurious findings that are driven by systematic differences in acquisitions rather than by true underlying effects. Our harmonization allows for our two datasets to be combined into joint analyses while preserving the underlying structure of associations with real-world outcomes.

Our study was focused on single-source unsupervised domain adaptation approaches, which might limit application in terms of applying the method to a different neonatal dataset. However, by utilizing reliable tissue segmentation maps from multiple neonatal databases, the proposed model can be extended to a multi-source domain adaptation pipeline (Mansour et al., 2008; Xu et al., 2018). Additionally, the latent based domain adaptation method was trained using the features at every layer of the decoding branch, without analyzing different combinations of the encoding-decoding layers. Future work will therefore aim to systematically evaluate our design choices via ablation studies. At the same time, we focused our work on investigating structural (*T*2w) datasets only, and in future we aim to extend this study to harmonize diffusion data as well.

## Data Availability Statement

The code developed for this study are available online on Github (https://github.com/irinagrigorescu). Imaging data collected for the dHCP are available in early 2021 at http://developingconnectome.org/. Requests for Data Sharing for the ePrime dataset should be made to the Chief Investigator of (Edwards et al., 2018) doi: 10.1136/archdischild-2017-313102.

## Ethics Statement

The studies involving human participants were approved by the National Research Ethics Committee (dHCP, REC: 14/Lo/1169; EPrime, REC: 09/H0707/98). Informed written consent was given by parents prior to scanning.

## Author Contributions

IG prepared the manuscript, implemented the code for the domain adaptation models and the analysis. LV participated in the implementation of the analysis code, the study design and interpretation of the results. AU assisted with data preprocessing, design of the study and interpretation of the results. DB performed preprocessing of the dHCP and ePrime datasets. LC-G developed MRI acquisition protocols for the neonatal dHCP datasets. CN participated in the study design and interpretation of the results. ADE and JVH are coordinators of the dHCP project. MM supervised all stages of the current research. MD conceptualized the study, supervised all stages of the current research and preparation of the manuscript. All authors gave final approval for publication and agree to be held accountable for the work performed therein.

## Conflict of Interest

The authors declare that the research was conducted in the absence of any commercial or financial relationships that could be construed as a potential conflict of interest.

## Footnotes

^1^http://www.developingconnectome.org/

^2^https://www.npeu.ox.ac.uk/prumhc/eprime-mr-imaging-177

^3^https://tools.npeu.ox.ac.uk/imd/
